# Supplementary figures and images for: DAnkrd49 and Bdbt act via Casein kinase Iε to regulate planar polarity in Drosophila
Source: PLoS Genet. 2020 Aug 4;16(8):e1008820. doi: 10.1371/journal.pgen.1008820 (PMC7402468; doi:10.1371/journal.pgen.1008820)

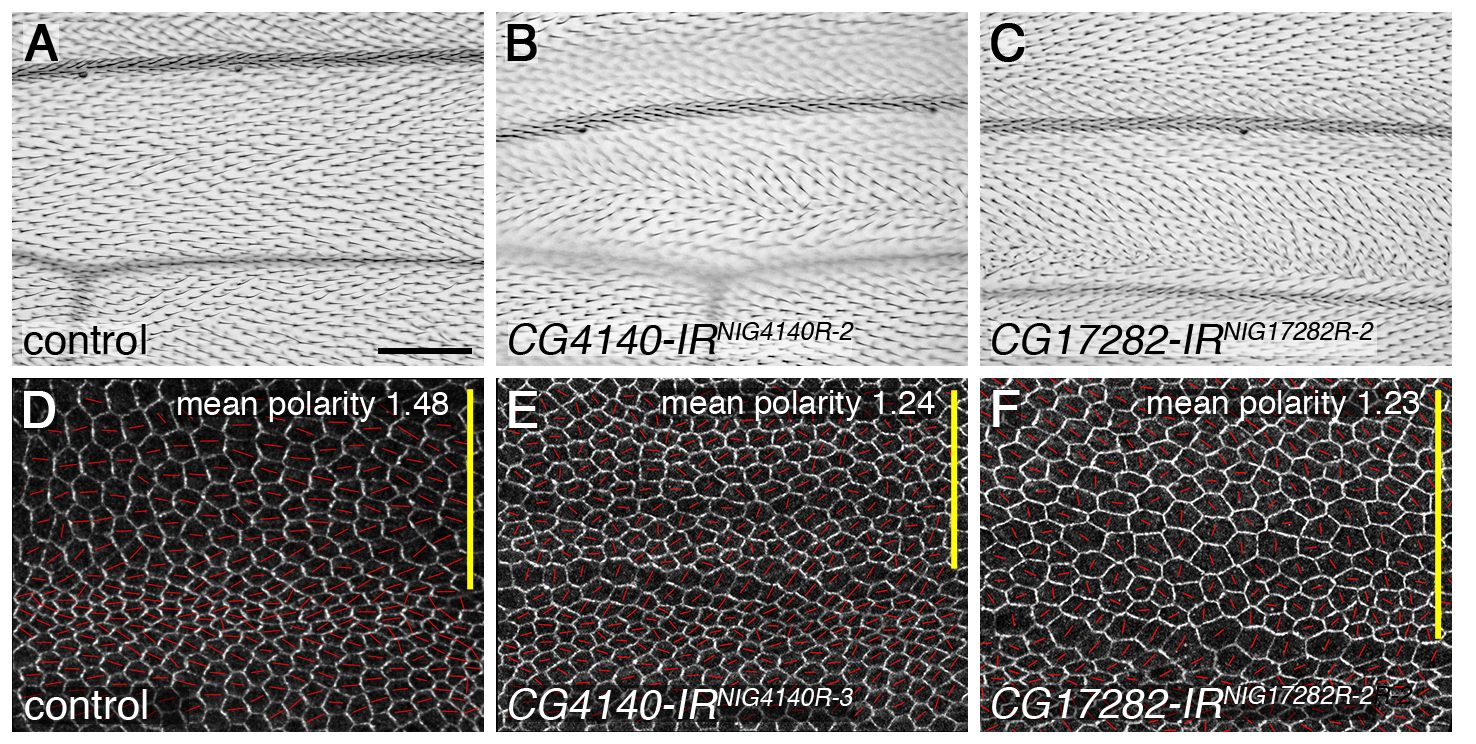

Supplement: S1 Fig — (A-C) Dorsal surface of adult male wings from control wild-type flies (A), or from flies expressing RNAi against CG4140/DAnkrd49 (B, NIG line 4140R-2), or CG17282/Bdbt (C, NIG line 17282R-2), under control of the ptc-GAL4 driver at 25°C, in the presence of UAS-Dcr2. Scale bar 100 μm. (D-F) Pupal wings expressing a control RNAi (D, VDRC line 39864, targeting Sik1, a gene unrelated to planar polarity), or RNAi against CG4140/DAnkrd49 (E, NIG line 4140R-3) or CG17282/Bdbt (F, NIG line 17282R-2), under control of the ptc-GAL4 driver (as in Fig 1E–1G),. Wings immunolabelled for Fmi. The yellow bar shows the ptc-GAL4 expression domain. The polarity nematic for each cell is shown as red lines. Polarity magnitude (length of red line) is quantitated in the ptc-GAL4 expression domain, and is reduced in wings expressing RNAi against DAnkrd49 and Bdbt (E,F) compared to control wings (D). The polarity angle is also more regular in wild-type wings. (TIF) [file pgen.1008820.s001.tif]

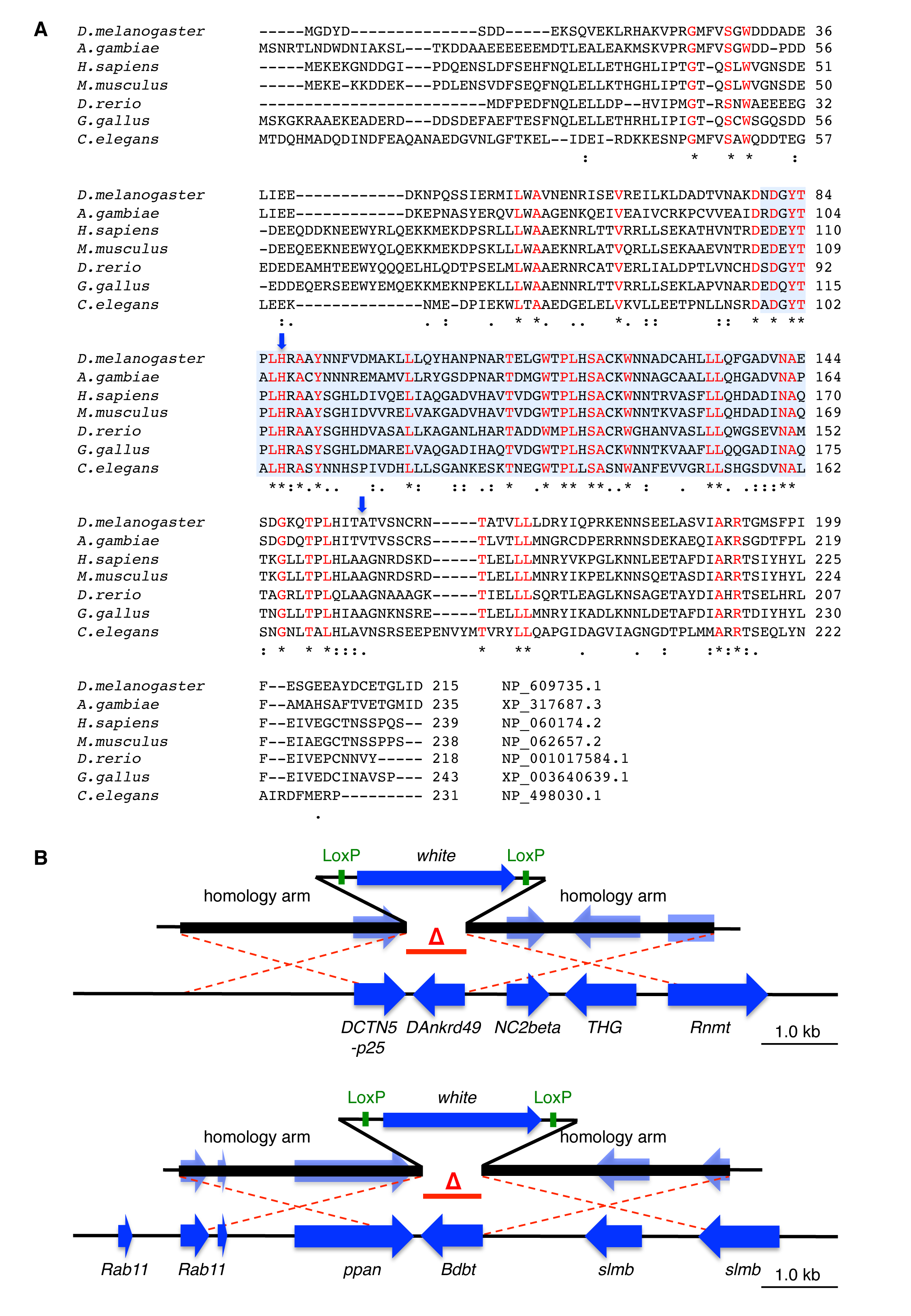

Supplement: S2 Fig — (A) Clustal alignment of DAnkrd49 with homologues. Asterisks (*) indicate conserved residues (also in red), colons (:) indicates conservation between groups of strongly similar properties, and full stop (.) indicates conservation between groups of weakly similar properties. Blue shading indicates the position of the ankyrin repeats, and blue arrows point to residues mutated in DAnkrd49l(2)35Be. (B) Diagram showing the open reading frames (blue) in the genomic regions surrounding DAnkrd49 (top) and Bdbt (bottom). Homology arms corresponding to approximately 3 kb on either side of the target gene were inserted into the pRK2 vector on either side of a white gene flanked by LoxP sites (green). Recombination between the pRK2 transgene and the genomic DNA (red dashed lines) results in the target gene being exchanged for white (Δ in red). Regions of the homology arms encoding open reading frames in pRK2 were sequenced, to ensure no additional mutations were introduced. (TIF) [file pgen.1008820.s002.tif]

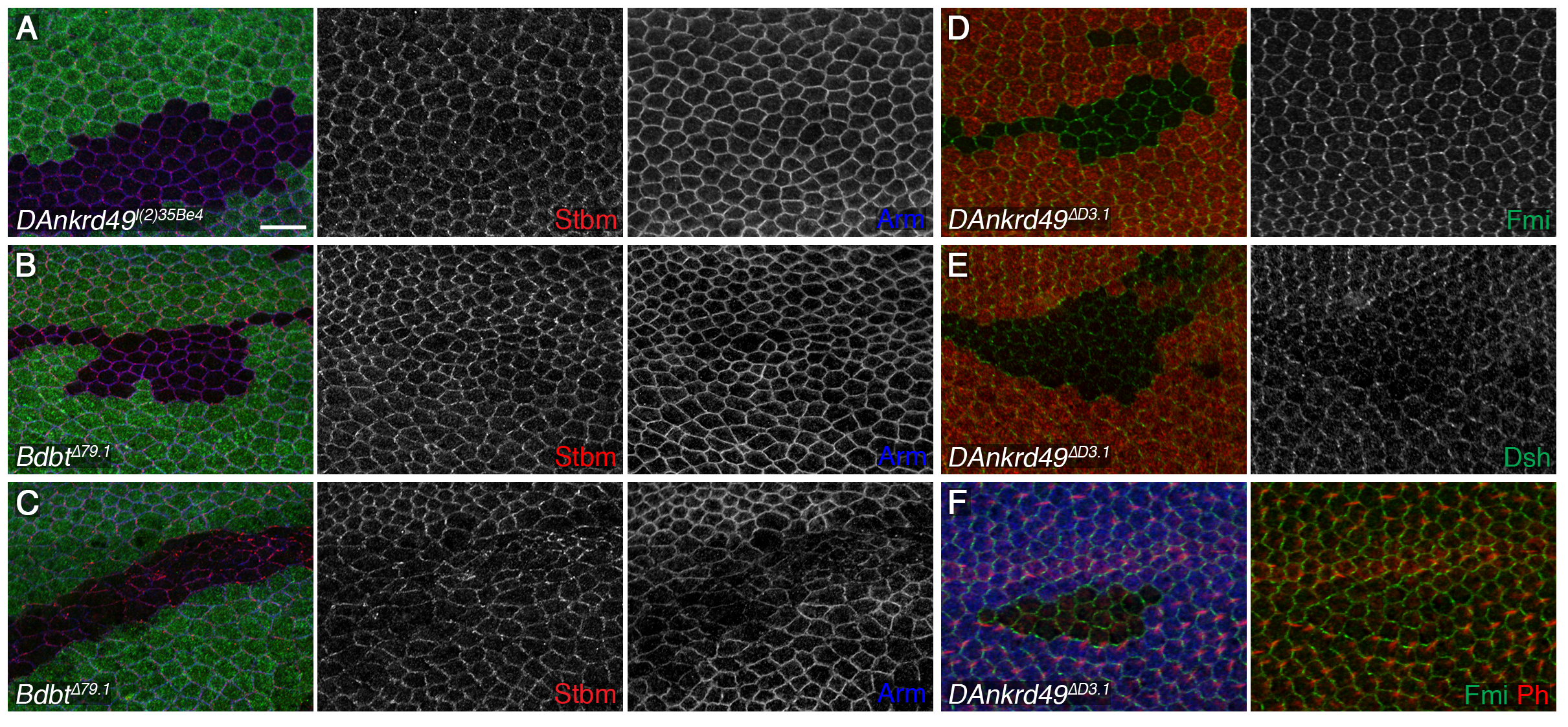

Supplement: S3 Fig — (A-C) 28hr APF pupal wings carrying clones of cells lacking DAnkrd49 (A, l(2)35Be4) or Bdbt (B and C, BdbtΔ79.1), marked by loss of β-gal (green). Wings immunolabelled for Stbm (red) and Arm (blue). (A,B) Arm staining is similar to wild-type in DAnkrd49 clones and some Bdbt clones. (C) In other Bdbt clones cells are abnormal, as seen by disrupted Arm localisation. Scale bar 10 μm. (D-F) 28hr APF pupal wings carrying clones of cells lacking DAnkrd49 (DAnkrd49ΔD3.1), marked by loss of β-gal (red in D and E or blue in F). Wings immunolabelled for Fmi (green in D and F), Dsh (green in E) and Phalloidin (red in F). (TIF) [file pgen.1008820.s003.tif]

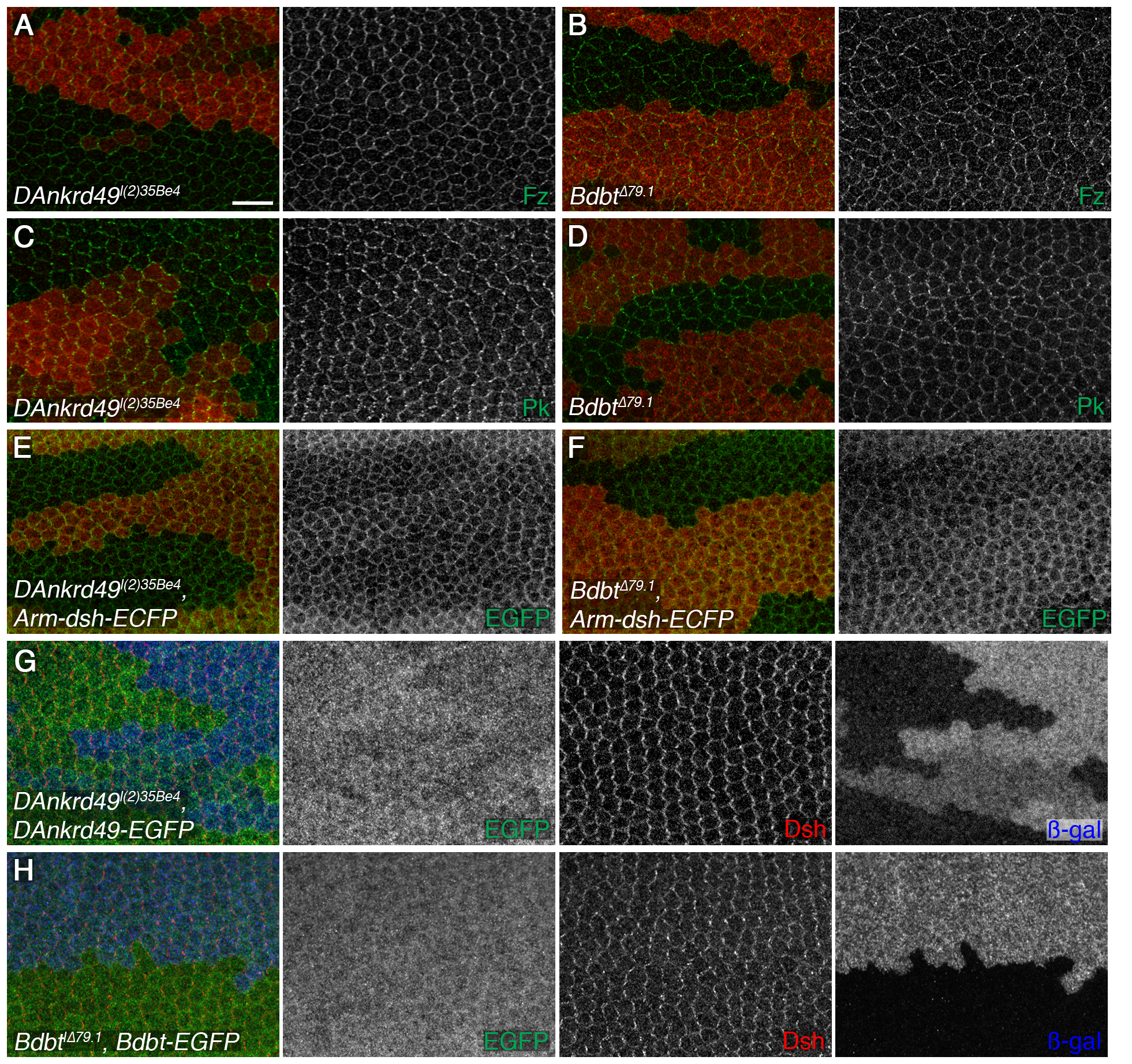

Supplement: S4 Fig — (A-D) 28hr APF pupal wings carrying clones of cells lacking DAnkrd49 (A,C, DAnkrd49l(2)35Be4), or Bdbt (B,D, BdbtΔ79.1) marked by loss of β-gal (red). Wings immunolabelled for Fz (green in A,B), or Pk (green in C,D). Scale bar 10 μm. (E,F) 28hr APF pupal wings carrying clones of cells lacking DAnkrd49 (E, DAnkrd49l(2)35Be4), or Bdbt (F, BdbtΔ79.1), in a background expressing one copy of Arm-dsh-ECFP. Wings immunolabelled for GFP (green), clones marked by loss of β-gal (red). (G,H) 28hr APF pupal wings carrying clones of cells lacking DAnkrd49 (DAnkrd49l(2)35Be4) in a background expressing ActP-DAnkrd49-EGFP (G), or clones of cells lacking Bdbt (BdbtΔ79.1) in a background expressing ActP-Bdbt-EGFP (H). Wings immunolabelled for GFP (green) and Dsh (red). Clones marked by loss of β-gal (blue). Dsh levels within the clone are similar to wild type in the presence of DAnkrd49-EGFP or Bdbt-EGFP (compare Dsh labelling inside and outside the clone tissue). (TIF) [file pgen.1008820.s004.tif]

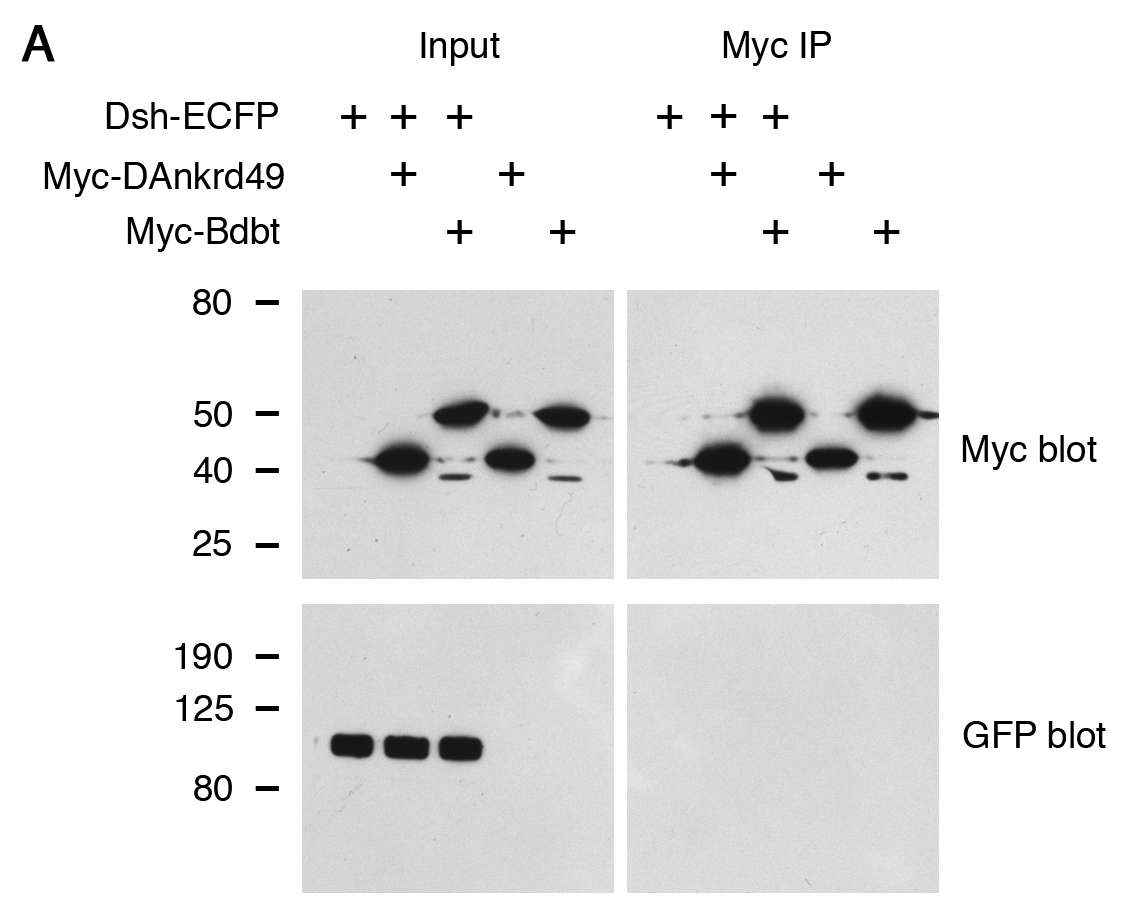

Supplement: S5 Fig — (A) Western blot showing co-immunoprecipitation experiment. S2 cells transfected with Dsh-ECFP and either Myc-DAnkrd49 or Myc-Bdbt. Immunoprecipitation with Myc antibody resin did not pull down Dsh-ECFP. (TIF) [file pgen.1008820.s005.tif]
