# Supplementary material for: DAnkrd49 and Bdbt act via Casein kinase Iε to regulate planar polarity in Drosophila
Source: PLoS Genet. 2020 Aug 4;16(8):e1008820. doi: 10.1371/journal.pgen.1008820 (PMC7402468; doi:10.1371/journal.pgen.1008820)
Supplement: S1 Table — Lines targeting CG4140 are 4140R-2 and 4140R-3, from the NIG RNAi collection, and 26396 and 109913 which are GD and KK lines respectively, from the VDRC. 4140R-2 and 4140R-3 are two insertions of the same target sequence, and target sequences overlap with lines 26396 and 109913. Lines targeting CG17282 are 17282R-2 and 17282R-3 from the NIG RNAi collection, and GD line 40059 from the VDRC. 17282R-2 and 17282R-3 are two insertions of the same target sequence, and target sequences overlap with line 40059. (DOCX) [file pgen.1008820.s006.docx]

| **Gene symbol** | **RNAI line** | **GAL4 driver** | **Temp** | **Adult wing phenotype** |
| --- | --- | --- | --- | --- |
| *CG4140/*  *DAnkrd49* | *4140R-2* | *MS1096-GAL4* | 25°C | Proximal swirls and multiple hairs. |
|  |  | *ptc-GAL4, UAS-Dcr2* | 25°C | Trichomes swirl towards the AP boundary. |
| *CG4140/*  *DAnkrd49* | *4140R-3* | *MS1096-GAL4* | 25°C | Proximal swirls and multiple hairs. |
|  |  | *ptc-GAL4, UAS-Dcr2* | 25°C | Lethal. |
| *CG4140/*  *DAnkrd49* | *26396* | *MS1096-GAL4* | 29°C | No phenotype. |
| *CG4140/*  *DAnkrd49* | *109913* | *ptc-GAL4, UAS-Dcr2* | 25°C | Trichomes swirl towards the AP boundary. |
| *CG17282/*  *Bdbt* | *17282R-3* | *MS1096-GAL4* | 25°C | Poorly viable. Escapers have proximal swirls and multiple hairs. |
|  |  | *ptc-GAL4, UAS-Dcr2* | 25°C | Distorted, uneven wings. |
| *CG17282/*  *Bdbt* | *17282R-2* | *MS1096-GAL4* | 25°C | Poorly viable. Wings distorted. |
|  |  | *ptc-GAL4, UAS-Dcr2* | 25°C | Trichomes swirl towards the AP boundary. |
| *CG17282/*  *Bdbt* | *40059* | *MS1096-GAL4* | 25°C | Poorly viable. Wings distorted. |
|  |  | *ptc-GAL4, UAS-Dcr2* | 25°C | Lethal. |
