# Supplementary material for: DAnkrd49 and Bdbt act via Casein kinase Iε to regulate planar polarity in Drosophila
Source: PLoS Genet. 2020 Aug 4;16(8):e1008820. doi: 10.1371/journal.pgen.1008820 (PMC7402468; doi:10.1371/journal.pgen.1008820)
Supplement: Attachment — Submitted filename: Strutt response to reviewers.docx [file pgen.1008820.s008.docx]

We thank the referees for recognising the valuable contribution of our work. Reviewer 1 says "The paper is clearly written and most of the experiments are well designed. The findings in this study provide an important contribution to understanding the molecular mechanism of PCP." Reviewer 2 says "The manuscript is well written and the data is of good quality and convincing. It provides new insights into the regulatory mechanism of core PCP proteins."

We have addressed most of the reviewers' comments, as indicated. However reviewer 1 has asked for additional cell culture experiments which are impossible for us to carry out at the moment. We have already been out of the lab for 11 weeks due to the Covid-19 crisis, and our institution has not yet given any indication as to when we will be allowed back into the lab - the last estimate from our Head of Department was August! - and there will certainly be restrictions on our ability to work even then. We think that the additional cell culture experiments, while interesting, are not necessary to support our conclusions, and we hope that you will consider our manuscript for publication as presented.

**Reviewer #1:** In this manuscript, Strutt et al. identified Bdbt and DAnkrd49 as novel planar cell polarity (PCP) proteins. The authors revealed that these two proteins physically interact in vitro and regulate each other’s protein levels in vivo. They also found that Bdbt and DAnkrd49 are essential for asymmetric localization of the core proteins in the Drosophila pupal wing and regulate overall levels and phosphorylation of Dsh. Further analyses provided evidence that Bdbt and DAnkrd49 act together to regulate Dco activity, which in turn phosphorylates Dsh during PCP establishment.

Overall, the paper is clearly written and most of the experiments are well designed. The findings in this study provide an important contribution to understanding the molecular mechanism of PCP. However, I think that the following points, especially the molecular mechanism on how DAnkrd49 regulates Dco activity, need to be clarified and/or corrected.

(1) A previous study by Fan et al. demonstrated that in the context of circadian rhythms, Bdbt physically interacts with Dco and positively regulates its activity. The authors and other groups previously reported that Dco phosphorylates Dsh in PCP signaling. These data taken together propose a model that Bdbt modulates Dco activity, which in turn regulates Dsh phosphorylation. In this study, the authors demonstrated that this regulatory pathway actually works during PCP establishment in the Drosophila wing and that DAnkrd49 is also implicated in this pathway through the regulation of Dco activity. However, it is unclear to me how DAnkrd49 contributes to the regulation of Dco activity. Obviously, one possible explanation is that DAnkrd49 regulates Dco activity through interaction with and stabilization of Bdbt. The authors should examine whether the two point mutations seen in the DAnkrd49l(2)35Be4 allele, which causes a reduction of Bdbt in vivo, affect the physical interaction between DAnkrd49 with Bdbt.

(2) This is related to point 1 above. It would be important to examine whether DAnkrd49, Bdbt and Dco can form a trimeric complex.

With regard to the above two points, we have not found direct binding between DAnkrd49 and Dco – specifically we did transfections into S2 cells, as in Figure 2C, but saw no pulldown. We therefore think it most likely that DAnkrd49 acts by stabilising Bdbt, which itself regulates Dco activity (as demonstrated by Fan et al). We don't show this negative result, but have now mentioned it as unpublished data in the discussion. We have now also discussed in the manucript how DAnkrd49 could contribute to regulating the activity of Dco via Bdbt.

It is likely that Dco, DAnkrd49 and Bdbt form a trimeric complex, unless their binding sites overlap. However, due to the Covid-19 situation we are unable to do new experiments at this time to test this, as we do not have access to the lab. Likewise, we are not able to test whether the point mutations in DAnkrd49 disrupt the physical interaction between DAnkrd49 and Bdbt.

However, we feel that the additional experiments would not materially alter the conclusions of the manuscript, and therefore hope that the reviewer will be happy to accept the revised manuscript as presented.

(3) In Figure1, control pictures should be provided for comparison.

We thank the reviewer for pointing out this omission. We have now provided control images for the panels in Figure 1 and Figure S1.

(4) In Figure 3A’, the control region on the bottom-left corner seems to be treated as a part of the mutant clone (because of no yellow line). If so, the values should be corrected.

The yellow line on Figure 3A' has been corrected, so that the bottom left corner is wild-type not mutant. However, the quantitation of the mutant tissue in this image did not include this wild-type region, so the values shown on the image are correct. Multiple images were also quantitated in Figure 3E.

(5) Page 7, line 147: “target genes” should be “target proteins”.

This has been corrected.

Reviewer #2: In this study, the authors identified two new regulators of drosophila PCP. They found Bdbt and dAnkrd49 bind to each other to regulate Dsh protein levels and Dsh phosphorylation via Ser/Thr kinase Dco. The manuscript is well written and the data is of good quality and convincing. It provides new insights into the regulatory mechanism of core PCP proteins. It is suitable for publication in PLoS Genetics after the following comments and suggestions have been addressed.

1. Does Ankrd49 directly bind to Dco, or it affects Dco indirectly through stabilizing Bdbt?

We have not found direct binding between DAnkrd49 and Dco in co-IP experiments – specifically we did transfections into S2 cells, as in Figure 2C. We therefore think it most likely that DAnkrd49 acts by stabilising Bdbt, which itself regulates Dco activity (as demonstrated by Fan et al). As this is negative data we have not included it in the manuscript, but we have mentioned the result in the Discussion as unpublished data. We have now also discussed in the manuscript how DAnkrd49 could contribute to regulating the activity of Dco via Bdbt.

2. Why is the phenotype mainly restricted to the proximal region of the wing? What is the normal expression pattern of Bdbt and dAnkrd49 in drosophila wing?

We do not fully understand why expressing the RNAi lines against Bdbt and DAnkrd49 using the *MS1096-GAL4* driver (Figure 1) gives phenotypes that are restricted to the proximal region of the wing. The published data shows that the *MS1096-GAL4* driver is expressed throughout the dorsal wing pouch.

However, it is important to note that loss of function clones for both genes give phenotypes **throughout** the wing and loss of function clones can be rescued by uniform mis-expression, suggesting that the genes are widely expressed and that there is no requirement for graded expression. Hence, we don’t think that the stronger proximal phenotypes with RNAi suggest that gene function is only required proximally.

We also know from published databases that *Bdbt* and *DAnkrd49* are expressed throughout wing development, but we do not know if their levels are uniform throughout the developing wing tissue. It is possible that their levels of expression are lower near to the wing hinge, and this could make them more sensitive to knockdown in this region.

3. Figure 4A, B, the rescue effect of DAnkrd49-EGFP and Bdbt-EGFP should also be shown by the trichome orientation.

Recent work (e.g. Strutt et al, 2016) has shown that trichome orientation is a relatively insensitive assay for detecting polarity defects: trichomes can still orient correctly even when core protein asymmetry is significantly reduced compared to a wild-type situation. We have therefore quantified core protein asymmetry in the rescued clones, as this is a more sensitive assay. This shows clearly that the mutant phenotype is rescued by DAnkrd49-EGFP and Bdbt-EGFP, and this result has been added to Figure 4.

4. Authors found it is difficult to verify whether loss of Dankrd49 or Bdbt reduces Stbm phosphorylation due to the limited mobility shift of Stbm on the SDS-PAGE. Is it possible to overexpress Fz to induce Stbm phosphorylation (as shown by Kelly et al. Cell Reports 2016) to make it more visible, then test the effect of Dankrd49 or Bdbt?

This is a good idea, but unfortunately our published experiments disagree with Kelly et al, and suggest that Fz does not regulate Stbm phosphorylation in vivo.

Kelly et al overexpressed Fz in tissue culture cells and saw an increase in Stbm phosphorylation. We liked this result, as it would fit a feedback model whereby Fz on one side of a cell boundary destabilised Stbm on the same side, contributing to sorting of complexes. However, we examined Stbm phosphorylation in pupal wing extracts in vivo, and found that it was constitutively phosphorylated, and that loss of Fz did not give a reduction in Stbm phosphorylation that was visible on SDS PAGE (see Strutt et al, 2019). Thus, our in vivo data suggests that the proposed experiment will not be helpful to us in seeing a mobility shift of Stbm in the in vivo context.

5. In mammalian cells, Dvl is also highly phosphorylated by CK1. Given the potential interaction between Ankdr49, CK1 and FKBP family members, does knockdown or knockout of Ankdr49 (or FKBP family members) in mammalian cells also affect Dvl phosphorylation and protein levels? Although it is not necessary for this paper, I would suggest this experiment because it will increase the interest and impact of the manuscript for readers working in mammalian system.

This would be an interesting experiment, but we believe it is outside of the scope of this manuscript (as indicated by the editor).

6. A discussion regarding the phenotype of increase of cell size (Figure 1) and lethality would be useful. Is it because of additional substrates of Dco?

We agree that the other phenotypes could be due to other substrates of Dco, for example Dco is known to regulate cell growth and survival via the Hippo signalling pathway, and also regulates Hh signalling and canonical Wnt signalling. However we also cannot exclude that Bdbt and DAnkrd49 also have other substrates in addition to Dco. We have now discussed this interesting point in the manuscript.
